# Open-source environmental data as an alternative to snail surveys to assess schistosomiasis risk in areas approaching elimination

**DOI:** 10.21203/rs.3.rs-2511279/v1

**Published:** 2023-01-27

**Authors:** Elise Grover, William Allshouse, Andrea Lund, Yang Liu, Sara Paull, Katherine James, James Crooks, Elizabeth Carlton

**Affiliations:** Colorado School of Public Health; Colorado School of Public Health; Colorado School of Public Health; Sichuan Center for Disease Control and Prevention; National Ecological Observatory network (NEON); Colorado School of Public Health; National Jewish Health; Colorado School of Public Health

**Keywords:** Schistosomiasis, geographic information systems, remote sensing technology, machine learning, prevention and control, China, infectious disease surveillance, snails, Oncomelania hupensis

## Abstract

**Background:**

Although the presence of intermediate snails is a necessary condition for local schistosomiasis transmission to occur, using them as surveillance targets in areas approaching elimination is challenging because the patchy and dynamic quality of snail host habitats makes collecting and testing snails labor-intensive. Meanwhile, geospatial analyses that rely on remotely sensed data are becoming popular tools for identifying environmental conditions that contribute to pathogen emergence and persistence.

**Methods:**

In this study, we assessed whether open-source environmental data can be used to predict the presence of human *Schistosoma japonicum* infections among households with a similar or improved degree of accuracy compared to prediction models developed using data from comprehensive snail surveys. To do this, we used infection data collected from rural communities in Southwestern China in 2016 to develop and compare the predictive performance of two Random Forest machine learning models: one built using snail survey data, and one using open-source environmental data.

**Results:**

The environmental data models outperformed the snail data models in predicting household *S. japonicum* infection with an estimated accuracy and Cohen’s kappa value of 0.89 and 0.49, respectively, in the environmental model, compared to an accuracy and kappa of 0.86 and 0.37 for the snail model. The Normalized Difference in Water Index (NDWI) within half to one kilometer of the home and the distance from the home to the nearest road were among the top performing predictors in our final model. Homes were more likely to have infected residents if they were further from roads, or nearer to waterways.

**Conclusion:**

Our results suggest that in low-transmission environments, investing in training geographic information systems professionals to leverage open-source environmental data could yield more accurate identification of pockets of human infection than using snail surveys. Furthermore, the variable importance measures from our models point to aspects of the local environment that may indicate increased risk of schistosomiasis. For example, households were more likely to have infected residents if they were further from roads or were surrounded by more surface water, highlighting areas to target in future surveillance and control efforts.

## Background

The water-borne disease, schistosomiasis, has been targeted for elimination in regions such as China, where decades-long control programs have led to major reductions in infections and morbidity (1). However, as transmission becomes more sporadic as a result of successful disease control programs, surveillance strategies also need to be recalibrated to allow e cient identification of pockets of on-going infection at fine spatial scales so that these areas can be targeted for treatment and transmission-blocking interventions.

Schistosomiasis is a di cult disease for surveillance due to its gradual onset, and its non-specific and intermittent symptoms such as abdominal pain, diarrhea and rectal bleeding (2). When left untreated, this can lead to a range of serious conditions including stunted childhood development and cognitive impairment, anemia, pulmonary hypertension, fibrosis of vital organs, and in the most serious cases, death (2). The slow and non-specific disease onset means infected individuals rarely seek care upon infection, and thus passive clinic and/or hospital-based surveillance, widely used for other infectious diseases, are not reliable ways to monitor infections. (Notably, some naïve individuals develop acute morbidity upon infection, due to an in ammatory reaction to the migrating schistosome (3). Acute schistosomiasis, or Katayama fever, can signal emerging infections, but reliance on acute case reporting alone will lead to misclassification of many areas with ongoing transmission (4).)

In China, surveys for the presence of the intermediate snail host and *S. japonicum* infections in snails have formed a key part of schistosomiasis surveillance strategies (5). *Oncomelania hupensis* is the only intermediate host species that is capable of passing *S. japonicum* to humans and other mammalian hosts in mainland China, making environmental conditions that favor snail habitat, as well as the distribution and abundance of *O. hupensis* across local ecosystems a practical and common focal point of local control programs (6). The significance of the ambient environment in the schistosomiasis transmission cycle is heightened by the fact that a single lifecycle involves two key timepoints when the developing parasite must survive in open water, moving quickly from a mammalian host’s feces to an intermediate snail host during the miracidia stage, and later swimming from an intermediate snail to a new mammalian host during the cercarial stage of the schistosomiasis lifecycle (2, 7, 8). Thus, a combination of several environmental conditions – including soil and vegetative health, the presence of fresh water, temperature, season and elevation – can all significantly impact the likelihood of snail habitation, the survival of the parasite, and the overall transmission potential of a given location (6, 8–10).

Despite the key role that snails play in the transmission of schistosomiasis, using them as surveillance targets is challenging due to the patchy and dynamic quality of snail habitats and the sparsity of snail infections. Identifying, collecting and testing snails for *schistosoma* infections is time-consuming and labor-intensive requiring surveying kilometers of transects, collecting thousands of snails and repeating surveys to account for seasonal fluctuations in snail populations (11, 12). Although infected snails are a necessary condition for mammalian schistosome infection to occur, they are often poor predictors of human infection risk (4, 11–13). For example, assessments conducted in Jiangsu, Yunnan and Sichuan Provinces between 2016 and 2017 did not find any *S. japonicum* infected snails from several million that were systematically identified, collected and tested during comprehensive snail survey efforts, despite having identified low to moderate levels of infection in humans and other mammalian hosts (14–16). Similarly, while the presence of intermediate snail hosts has been broadly correlated with human and livestock infection in some instances (17, 18), the transient, impermanent nature of snail habitats can also make them an inconsistent predictor of human infection risk and an unreliable target for schistosomiasis surveillance (12).

As a result, assessments of schistosomiasis transmission environments have increasingly relied on measures of environmental characteristics, often using remote sensing in combination with geospatial analyses. There is a considerable body of literature demonstrating the use of such measures to estimate environmental suitability for snail habitation (e.g., 6, 10, 18–20), which can theoretically be used to highlight potential schistosomiasis hotspots. For example, the Normalized Difference Water Index (NDWI), an indicator of surface water presence, has been found to be a significant, reliable predictor of snail habitat locations when applied at a 250-m resolution to a ~ 1200 km^2^ area with distinct dry and wet seasons within KwaZulu-Natal province, South Africa (21).

Meanwhile, in more densely vegetated areas of China, other environmental characteristics have been identified as strong predictors of snail habitats, including elevation, humidity, annual average precipitation, vegetation type, the normalized difference vegetation index (NDVI) and distance to the nearest waterbody (6, 10, 19). There is considerable variability in key predictors of snail habitat when comparing across different ecosystem types, even within regions of China. For example, one study spanning several hundred kilometers along the Yangtze River in Anhui Province indicated that distance to the nearest river was the most important predictor of snail habitats in marshland ecosystems, whereas 100-m resolution summaries of mean temperature and annual precipitation were the most important predictors within the hilly regions of the Province (19). Notably, across smaller geographic areas, factors like ecosystem type and climatic conditions would not be expected to vary meaningfully, highlighting the ongoing need to identify metrics that can predict schistosomiasis at fine spatial scales.

Fewer studies have demonstrated the use of environmental characteristics to directly predict human schistosomiasis risk (9, 12, 22). A recent study in Senegal found that measures of vegetation and water contact area were better predictors of *S. haematobium* reinfection in children in a highly endemic region than measures from snail surveys (12). Similarly, studies of *S. japonicum* infection in China have found measures of vegetation and proximity to rivers were predictive of human infection clusters (9, 22). In all three studies, the models were designed to identify infections at the village scale. Further research is needed to identify higher resolution environmental proxies of human infection in low transmission settings. Moreover, localized investigations of the suitability of different environmental measures for predicting human infection across a range of settings are needed, as snail habitat preferences, suitability and transmission risks may vary substantially from ecosystem to ecosystem (22–25).

As China approaches schistosomiasis elimination goals, the perceived payoff of comprehensive infection and snail surveys will decrease, making it likely that resources will be diverted to other priorities in the coming decades. In order to avoid a resurgence in schistosomiasis, it is crucial that cost-effective, low labor surveillance techniques are developed that can be used to pinpoint, at fine geographic scales, areas of high infection risk in areas approaching elimination. Precision risk mapping can enable targeting of resources to high-risk areas for testing, treatment or transmission-blocking interventions. The proliferation of high-resolution, open-source geospatial data products offer an opportunity to develop new methods for mapping schistosomiasis risk in areas where control programs have reduced but not fully eliminated schistosomiasis.

Our study aimed to determine whether open-source environmental data that is freely available and less time- and labor-intensive to collect than snail survey data can directly predict household *S. japonicum* infection distribution, with a similar or improved degree of accuracy as data obtained during snail surveys. To do this, we developed and compared two models for predicting household *S. japonicum* infection among rural farming communities in Sichuan Province, China. In our first model, we used geocoded snail survey data to build a set of predictors and determine how well the proximity and density of snail habitat relative to the location of the home predict household *S. japonicum* infection status. In the second model, we drew on freely available, open-source environmental data to create a set of measures characterizing local surface water and vegetation density in the area surrounding the home in the months prior to infection surveys. By comparing the ability of these two models to predict fine-scale geographic patterns of human *S. japonicum* infection, our study provides valuable information on the utility of each of these surveillance techniques for identifying potential high and low risk households in communities where low levels of persistent *S. japonicum* infection are obstructing elimination goals. Furthermore, by comparing the relative predictive performance of a range of measures of snail habitat and environmental conditions at different proximities to the home, this study sheds light on those characteristics of the local environment that can best be leveraged for predicting household *S. japonicum* infection risk and targeted in local prevention and control efforts.

## Methods

### Setting and village selection

**Figure 1:**
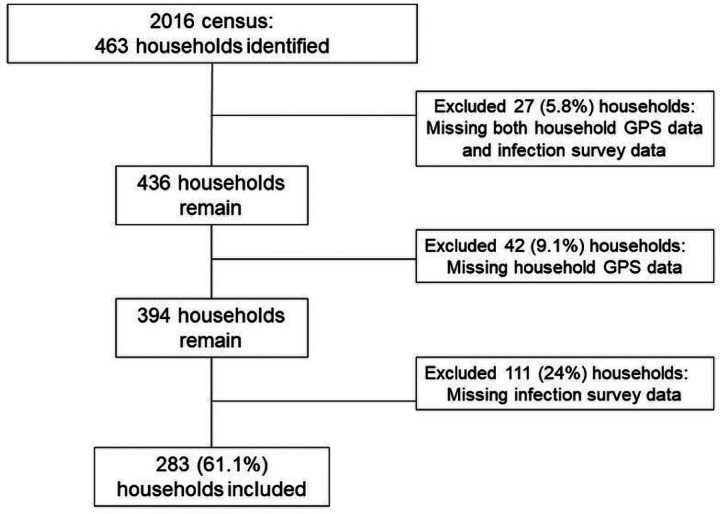
Depiction of household exclusion and inclusion.

This study was conducted in 2016 in two counties located in the hilly regions of Sichuan Province, China. County surveillance records were used to select ten villages at high risk of reemergent or ongoing *S. japonicum* transmission. We conducted a census in each village, attempted to geocode the location of all households using handheld Global Positioning System (GPS) devices, and surveyed each household for *S. japonicum* infection, as described below. The number of households in the selected villages ranged from 19 to 75, with between 50 to 250 residents residing in each village at the time of data collection. We restrict this analysis to households for which: a) GPS coordinates were successfully recorded, and b) at least one household resident was tested for *S. japonicum* infection during the 2016 infection surveys. Of the 463 households identified during the census, a total of 283 households (61.1%) had both GPS and infection survey data and are therefore included in this analysis. See Figure 1 for details on household exclusion and inclusion.

### Data collection and sources

Human infection data were collected in July 2016 as a part of ongoing research efforts in the region assessing persistent schistosomiasis hotspots. All village residents over the age of five were invited to participate in the study. Each participating individual was asked to provide three stool samples on consecutive days. All samples were labelled with the date of collection and participant ID numbers and stored in a cooler or cool room (ideally <10°C) until they could be transported to the central laboratory for processing. Samples were examined using the miracidium hatching test, following standard protocols (26). In brief, for each sample, 30 grams of stool was suspended in water (pH range of 6.8 – 7.2), strained to remove large particles (80 head nylon mesh), strained again to retain the remaining solids (280 head nylon mesh), and then suspended in water at room temperature (28 – 30°C). At 2-, 4- and 8- hours after suspension, the samples were examined for the presence of miracidia for at least 2-minutes each time. An individual was considered positive if any of the three hatch tests were positive.

The habitats of *O. hupensis* snails in the present study were determined during a national survey on *O. hupensis* that was conducted in 2016. Snail habitats were first identified by trained professionals from county anti-schistosomiasis control stations using historical records that date back to the 1950s. Historical habitats were digitized by global positioning and geographic information systems (GIS). Surveys of *Oncomelania* snails were then conducted in the field via transect walks between the months of April and October 2016 using standard systematic sampling methods (27). Briefly, each historic, existing or suspected snail environment was divided into sampling frames set every 5–10 meters along the water line for linear features (e.g., ditches, rivers, etc.) and every 5–10 meters along the periphery of polygon features (e.g., flooded paddy fields, ponds, etc.), with parallel lines extending from each to form a set of sampling frames of between 25 – 100 m^2^ covering each site. The majority of existing or suspected sites were characterized by shallow, stagnant or moving water (e.g. a stream, pond, rice paddy or irrigation ditch), as these conditions are the preferred habitat of the amphibious freshwater *O. hupensis* snails (28). For each site, ~20% of the sampling frames were randomly selected to be investigated on foot for the presence of snails. The digitized maps were updated using handheld GPS devices to document present and absent habitat locations, shapes, and whether and how historic habitats had been destroyed or changed (e.g., land use change via urbanization). For each site, the environment type was recorded as either a polygon or line feature in the dataset. Polygons most frequently represented rice paddy fields in the area, though they occasionally represented other habitat sites such as a small pond, a dry field or a beach. Meanwhile, line features most commonly represented dirt or concrete irrigation ditches used for flooding rice paddies, though it could also correspond with streams or other narrow waterways. For the purposes of this analysis, all polygons are referred to as “ fields”, while all lines are referred to as “ditches”.

Data on waterbodies, waterways and roads in Sichuan Province, China were obtained from the OpenStreetMap project, downloaded on November 11^th^, 2021 (29). The OpenStreetMap project draws on local communities of mappers to build a knowledgeable database detailing roads, waterways, transportation and other built and natural environment features (30). OpenStreetMap Contributors use aerial imagery, handheld GPS devices, and field maps, both to generate the data and to verify the accuracy of the open data on a regular basis (30). The roads data from the OpenStreetMap project ranges from national freeways and motorways down to gravel tracks and paths, while the waterways and waterbodies included permanent water features such as large rivers, streams, canals, lakes and reservoirs. Details on the OpenStreetMap data used in this analysis can be found at: https://download.geofabrik.de/osm-data-in-gis-formats-free.pdf (31).

Elevation Data was obtained from the Earth Observation Research Center Japan Aerospace Exploration Agency’s (JAXA EORC) Advanced Land Observing Satellite (ALOS) global digital surface model, which has a horizontal resolution of approximately 30 meters (32). To calculate indices of vegetation and waterbody coverage, the U.S. Geological Survey’s (USGS) Earth Resources Observation and Science (EROS) Center’s image library from the Landsat Satellite 8 – Collection 1 was accessed from the USGS Earth Explorer website (https://earthexplorer.usgs.gov/) to obtain data on surface reflectance bands 2 – 5, as well as the QA band (33). The Landsat-8 satellite repeats its orbital pattern every 16-days (34), resulting in a total of 12 available observations across 2016 that occurred prior to our July infection surveys, which were downloaded for use in this study. The National Aeronautics and Space Administration’s (NASA) pre-processed Moderate Resolution Imaging Spectroradiometer (MODIS) Terra satellite imagery database was also accessed to obtain 250-meter resolution data on vegetation at 16-day intervals at all available timepoints in 2016 prior to the infection surveys (35).

### Variable definitions and generation

#### Outcome variable

*S. japonicum* infection survey results from the ten study villages were aggregated to the household level and spatially joined to the geographic location of the home. To avoid issues with multicollinearity resulting from residents of the same household having the same values for all environmental predictors used in this analysis, the outcome was a binary measure of household infection status, with “0” indicating no infections detected among participating household members, and “1” indicating that one or more household member tested *S. japonicum* positive. Each household was represented in the geospatial dataset as a point feature.

### Explanatory variables

#### Model 1: Snail survey data

Using the snail survey data collected in 2016, predictors were generated to assess how the household’s position in relation to surrounding snail habitats influence household-level *S. japonicum* infection risk (Table 1). The geocoded snail habitat data was divided into two categories: present snail habitat sites, and absent snail habitat sites. Present snail habitat sites were those sites where one or more snails were identified during the survey period, while absent snail habitat sites were those where snails were not found during the 2016 survey. The data were further grouped into “ditches” (i.e., line features deemed suitable for snail habitation) and “ fields” (i.e., polygon features deemed suitable for snail habitation), resulting in four snail habitat categories: present ditches, present fields, absent ditches, and absent fields.

For the snail survey data models, present sites are those where at least one snail was found, while absent sites are those where no snails were found during the 2016 snail surveys. For the environmental data models, NDVI and NDWI were calculated using Landsat-8, Collection 1 satellite data collected on January 23rd, February 8th, and April 28th (dates where there was < 30% cloud cover). Pre-processed EVI data from NASA’s Moderate Resolution Imaging Spectroradiometer (MODIS) Terra satellite was averaged across a total of 12 observations occurring at 16-day intervals between January 1st and July 10th, 2016.

Using ArcGIS Pro software (36), three different buffer sizes (0.25, 0.5 and 1.0 kilometer (km) radius length) were generated and applied to each household location using the “Buffer” analysis tool. These buffer radius lengths were defined such that the largest buffer (1 km) generally spanned the entire village area for a centrally located household, whereas the smallest buffer (0.25 km) spanned the immediate surroundings of a given household. The “Summarize Within” analysis tool was used to calculate the total length (km) of present ditches and absent ditches that fell inside each household buffer area. This step was repeated for the present fields and absent fields, calculating the total area of fields (km^2^) that were encapsulated by each household buffer. The “Near” analysis tool was then used to calculate the geodesic distance (in meters) between each household point and the nearest present ditch, absent ditch, present field, and absent field. The “Join Field” data management tool was used to join all of the newly created variables summarizing the length and area of ditches and fields into a single table, which was then exported to the project’s geodatabase for use in R. Including separate measures for the distance to the nearest ditch and nearest field, as well as the total length and area of ditches and fields within a given buffer area helped us determine whether these features varied in their relative predictive capacity or in their respective spatial scales (i.e. buffer sizes) of influence.

#### Model 2: Open-source Environmental data

Open-source environmental and remotely sensed data were compiled to create a geospatial dataset containing a range of hypothesized environmental (built and natural) predictors of household *S. japonicum* infection. Potential environmental predictors were selected if they were 1) previously identified or hypothesized in the literature to serve as predictors of schistosomiasis infection or snail habitat sites; and 2) made publicly available at a 250-meter resolution or finer for the entire study area. Most of the predictors represented natural features of the environment (e.g., waterbodies, elevation, vegetation indices, etc.). Human-made environmental features like roads were also included, as the relative remoteness or connectedness of a given household was hypothesized to be a factor associated with schistosomiasis infection status. Roads and waterways from the OpenStreetMaps project were included as line features, while waterbodies were water features coded as polygons. The “Near” Analysis tool was then used to calculate the geodesic distance (km) between each household and the nearest road, waterway, or waterbody.

Prior studies have suggested that elevation is negatively associated with the presence of *O. hupensis* snails (17, 37). We used 30-meter resolution elevation data from JAXA EORC’s ALOS satellite to extract the elevation (m) value that corresponded with each household point location.

The presence of either water or vegetation can provide opportunities for water contact and have the potential to impact human infection risk. In this study, we use the NDWI (38) to estimate water content across the study area, and the NDVI (39) and the Enhanced vegetation index (EVI) (40) to describe vegetation health and density in the study area. The NDWI identifies water features and distinguishes them from soil and vegetation surfaces (38). The NDVI index is chlorophyll-sensitive and provides a measure of crop and vegetation health, while the EVI is more sensitive to canopy variations and performs particularly well in high biomass regions (41). As such, the two vegetation measures complement each other and are frequently used jointly in vegetation studies (41). Whereas NDWI and NDVI were calculated using data from the Landsat-8, Collection 1, pre-processed EVI data at 250-meter resolution was downloaded directly from NASA’s MODIS data library (35).

There was a total of 12 Landsat-8 satellite images collected between January and July of 2016, all of which were examined and processed to remove all medium to high confidence clouds, cloud shadows or other sources of terrain occlusion. To do this, the QA band files made available by USGS for each of the Landsat-8 observations (34) were used in ArcGIS Pro with the “Remap” raster function, to recode all grid cells corresponding with terrain occlusion as “No Data”, while all clear or low confidence cloud cells were set to equal 1. The “Clip” Raster function was then used to remove all cells obscured by cloud cover from each corresponding Landsat-8 image. Overall, cloud cover was high between January 2016 – July 2016 over the study area, ranging from 9.97–100%, with an average cloud coverage of 66.13% across the 12 satellite observations made within that period. To assess the extent to which the removal of cloud cover and terrain occlusion would result in missing data for each household at each time point, the “Raster to Points” tool was used to convert the cloud-corrected satellite data to a grid of points, and the “Extract Multi Values to Points” geoprocessing tool was used to extract the data corresponding to each household’s point location. A total of 3 of the 12 Landsat-8 collections had < 30% cloud coverage and had cloud-corrected data available for between 98.5–100% of households. The remaining 9 collections had cloud cover ranging from 33–100%, which resulted in data for between 0–65% of households. As such, we restricted our use of the Landsat data to the 3 collections with < 30% cloud coverage (collected on January 23rd, February 8th, and April 28th of 2016).

Using ArcGIS Pro’s “Raster Calculator” Image Analyst tool, the NDWI on January 23rd, February 8th and April 28th were each calculated from the cloud-corrected Green and near Infrared (NIFR) Landsat Surface Reflectance bands (bands 3 and 5 in Landsat-8, respectively), using the following formula developed by McFeeters (1996) (38):

$$\text{NDWI}=\frac{\left(\text{Gree}n-\text{NIFR}\right)}{\left(\text{Green}+\text{NIFR}\right)}$$

NDVI on January 23rd, February 8th and April 28th was calculated from the cloud-corrected Red and NIFR Landsat Surface Reflectance bands (bands 4 and 5 in Landsat-8, respectively), using the following formula (39):

$$\text{NDVI}=\frac{\left(\text{NIFR}-\text{Red}\right)}{\left(\text{NIFR}+Red\right)}$$

The “Mosaic to new Raster” tool was then used to calculate an estimate of the average NDWI and NDVI across the three time points with sufficient data coverage.

EVI is calculated based on Blue, Red and NIFR Reflectance bands, as well as a soil adjustment factor (L), and two coefficients (C_1_ and C_2_) used to correct for aerosol scattering, as shown in the following formula (39, 41):

$$EVI=\left(1+L\right)*\frac{\left(NIR-Red\right)}{\left(\text{NIFR}+\left(\text{C}1*\text{Red}\right)-\left(\text{C}2*\text{Blue}\right)+L\right)}$$

All 12 of the pre-processed, cloud-corrected EVI measures at 16-day intervals for the period between January – July 2016 were downloaded from NASA’s MODIS Terra Satellite Imagery database (35), and were joined into a single, average EVI measure using the Mosaic to new Raster” tool in ArcGIS Pro.

Each of the final NDWI, NDVI and EVI measures were converted to a grid of points using the “Raster to Multi Points” tool. As was done for the snail data, three different buffers sizes were generated around each household point (0.25, 0.5 and 1.0 km radius), and the “Summarize Within” analysis tool was then used to calculate the average NDWI, NDVI and EVI in the 0.25, 0.5 and 1 km area surround each household. See Table 1 for a summary of all predictors included in the environmental predictors model.

### Analysis

To assess the predictive capacity of snail survey and environmental data to predict household *S. japonicum* infection status, and to compare the predictive performance of the model constructed using snail survey data to one that exclusively used open-source environmental data as predictors, a Random Forests (RF) machine learning approach was used. After the snail habitat dataset and the environmental predictors dataset were each generated in ArcGIS Pro, the RArcGIS Bridge from the ‘arcgisbinding’ R package was used to facilitate an easy transfer of data between ArcGIS and Rstudio for the RF analysis (42). Each dataset was split 75/25 for training and validation, respectively. For each training dataset, we oversampled the minority class to correct for class imbalance in our outcome variable (13.8% of households were *S. japonicum* positive). In total, three different balanced training datasets were generated for the snail data, and three for the environmental data, yielding a total of six balanced datasets that were used for RF model training. This approach allowed us to assess the stability of model performance metrics and variable importance rankings in light of our oversampling approach. The ‘caret’ package in R was used to perform a 10-fold cross validation process to tune each model, helping to determine the optimal maximum node size to use and the number of variables to try at each branch. For each RF model, we specified 5000 trees per forest, as a high number of trees is recommended to help stabilize variable importance rankings (43).

The reserved validation data was used to test each model and calculate performance statistics (accuracy, Cohen’s kappa statistic, receiver operator curve (ROC) area under the curve (AUC), sensitivity, specificity, positive predictive value (PPV) and the negative predictive value (NPV)). To compare performance between models, the best model was defined as the one with the highest kappa value, followed by accuracy and ROC AUC, respectively. The kappa statistic was selected as our main metric for indicating model performance because our reserved validation datasets had a high degree of class imbalance (13.8% of households were *S. japonicum* positive), and the kappa statistic was developed to help correct for bias related to over-rewarding the prediction of the majority class (44). Model accuracy was also compared to the No Information Rate (NIR), which indicates what the accuracy that would be expected to be if the majority class were predicted every time (NIR = 0.859). A high NIR value results when there is a high degree of class imbalance for the outcome of interest, as was the case in this study. Finally, in the event of a tie in the kappa and accuracy of two models, the ROC AUC was used to select a final, top performing model.

To determine which predictors were the most influential in predicting *S. japonicum* infection status in our models, the mean decrease in accuracy (MDA) values of predictors were visualized in variable importance plots for each model. For each of the three environmental data models and three snail data models, the top ten predictors indicated by the model’s MDA plots were given a score of 10 to 1 (10 being the score of the top predictor). Variable scores were then summed across the three models to create a three-model summary score of 0 to 30, 30 being the highest score possible, while a score of 0 indicates that the variable was never ranked among the top ten predictors. Simple logistic regression models and lowess plots were examined to determine the direction of association between household *S. japonicum* infection status and each predictor.

### Prediction mapping

Using the top performing RF model, a map of the predicted probability of *S. japonicum* infection across the entire study area was generated. Within ArcGIS Pro, the “Raster to Point” tool was used to generate a grid of points covering the entire study area surface. The grid dataset was then exported to R using the RArcGIS Bridge to calculate the predicted probability of infection at each point across the study area. These predicted probabilities were added to the grid dataset and exported back to ArcGIS Pro for mapping. Finally, the “Point to Raster” tool was used to transform the predicted probabilities into a raster surface, using the “Mean” method for the cell assignment type. All analyses were conducted in ArcGIS Pro 2.8.3 and Rstudio version 4.1.2 (36, 42).

## Results

**Figure 2:**
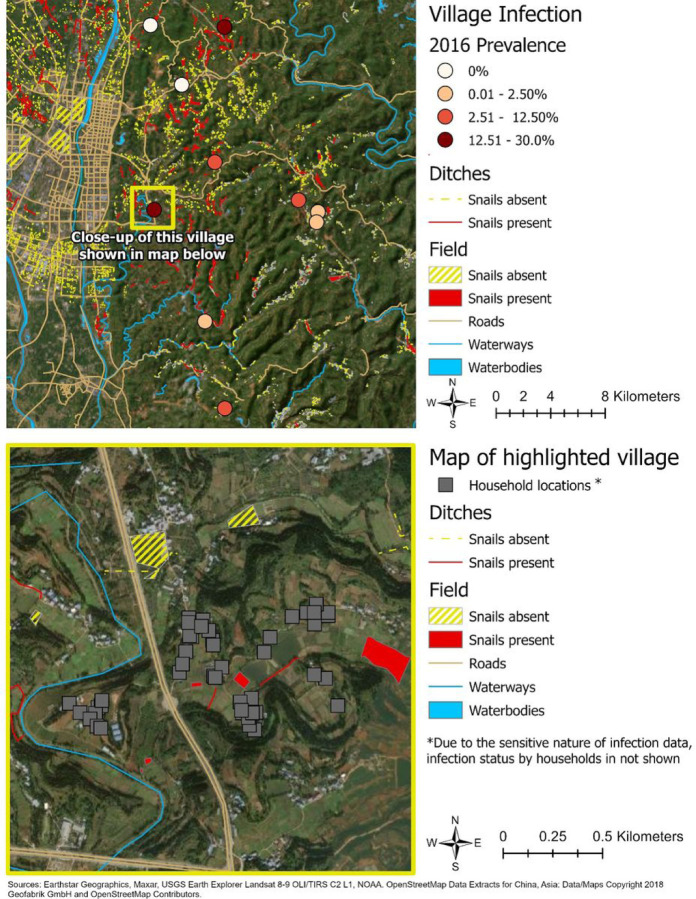
Map of the study villages.

Village-level *S. japonicum* infection prevalence (n=10) ranged from 0% to 27.1%, while the number of infections per household ranged from 0 to 3, with a mean of 0.16 (Standard Deviation (SD)=0.44) infections per household across the 283 households included. A total of 4,896 historical or current snail habitat sites were identified in the study area, of which 1,092 (22.30%) were found to contain one or more snails. None of the snails identified during the snail surveys were found to be infected with *S. japonicum*. In total, 1,485 (30.33%) of the surveyed sites were categorized as fields. Within 1 km of the home, the total area of fields (present or absent) ranged from 0 to 0.19 km^2^, while the average was 0.06 km^2^ (SD=0.06) for absent fields, and 0.04 km^2^ (SD=0.06) for present fields. A total of 3,413 (69.7%) sites were categorized as ditches. The total length of ditches within 1 km of the home ranged from 0 to 7.31 km long, with an average length of 1.74 km (SD=1.70) for present ditches, and 2.22 km (SD=1.35) for absent ditches. Figure 2 shows the snail survey data and village prevalence in the study area and an example of the geographic distribution of household infections in relation to the snail habitat sites within one village.

On average, the homes in our study villages were located closer to a road (0.36 km) than to a waterbody (2.11 km) or waterway (3.02 km). The mean elevation of households in the study villages was 573 m. Surface water in the area surrounding the home was generally low. NDWI values can range from −1 to 1, with a value of < 0 indicating a surface with little to no water content, though a threshold of >0.3 has been proposed as a reasonable value to use for identifying waterbodies (45). In our study, the mean NDWI within 1 km of the home was −0.19 (SD=0.01). Similarly, the NDVI and EVI range from −1 to 1, with lower values indicating more barren landscapes. Values lower than 0.1 for NDVI represent low vegetation areas (e.g. rocks, sand or snow), while values greater than 0.6 corresponds with temperate and tropical forests (46). For the EVI, values between 0.2 and 0.8 are generally used to indicate healthy vegetation (47). The average NDVI and EVI within 1 km of the home was 0.18 (SD=0.02) and 0.40 (SD=0.02), respectively. Table 2 provides summary statistics for the household predictors included in this analysis.

### RF Model Performance

**Figure 3:**
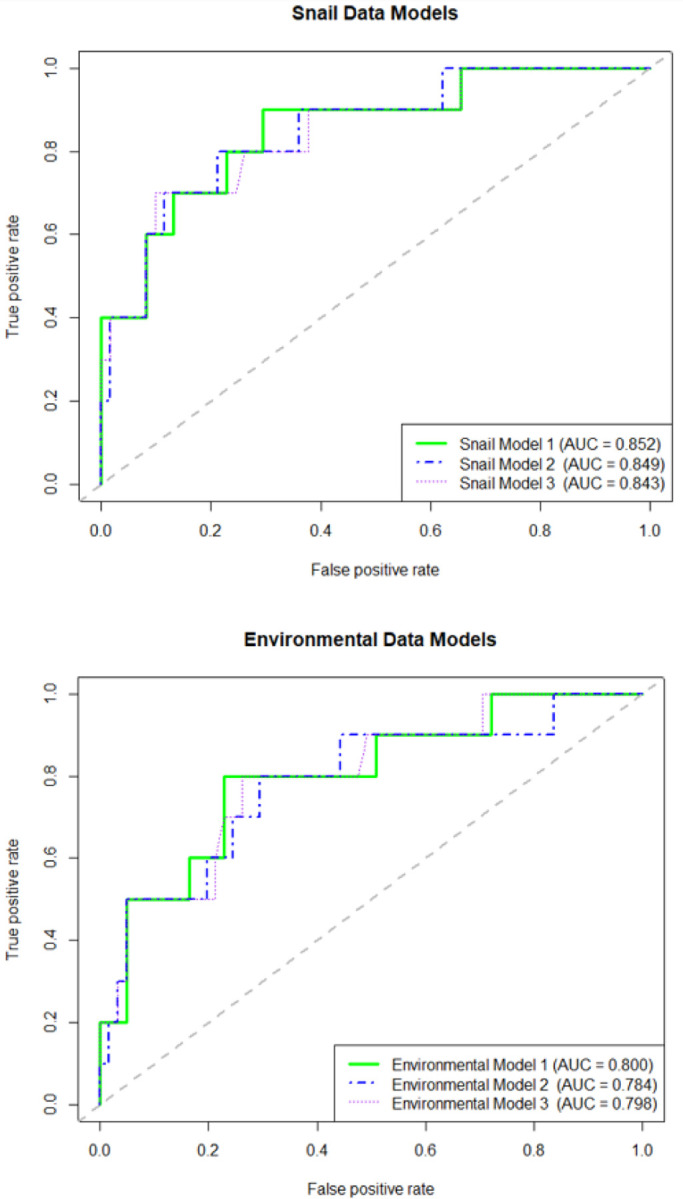
Receiver Operating Characteristics (ROC) Area Under the Curve (AUC) for snail and environmental data models.

The snail data models were outperformed by the open-source environmental data models using each model’s Cohen’s kappa, accuracy, and ROC AUC as the main metrics to gauge model performance (Table 3, Figure 3). Despite being outperformed in all other metrics, the ROC AUC of each snail model was higher than that of the environmental data models, with the best performing snail model producing a kappa, accuracy and AUC of 0.37, 0.86 (95% CI: 0.76 – 0.93) and 0.85 respectively. nevertheless, the accuracy of the best snail model was the same as the NIR of 0.86, which indicates what the accuracy would be expected to be if the majority class were predicted every time. According to the guidelines laid out by Landis & Koch (1977) on how to interpret the kappa statistic, their benchmarks suggests that the kappa values in our snail models (0.332 – 0.365) all suggest a “Fair” predictive capacity (0.21 – 0.40) (44). The sensitivity (the ability to predict a true positive) and the PPV (the odds of a true infection when infection is predicted) were low for the snail models, with a sensitivity of 0.4 and a PPV of between 0.44 – 0.5 for all snail models.

The kappa statistic and accuracy of the environmental models indicated strong predictive performance. The accuracy of all three open-source environmental data models was 0.89, slightly higher than the NIR of 0.86. The kappa statistic for all three environmental data models was 0.49, indicating the predictive capacity of the environmental models was “Moderate” (0.41 – 0.61) when using the Landis & Koch (1977) benchmarks (44). The ROC AUC for the environmental models ranged from 0.78 – 0.80. While the sensitivity and PPV for the environmental predictor models was still relatively low (sensitivity: 0.5; PPV: 0.63), the specificity of the models (0.95) and NPV for all three models (0.92) were very high.

### Variable Importance

**Figure 4:**
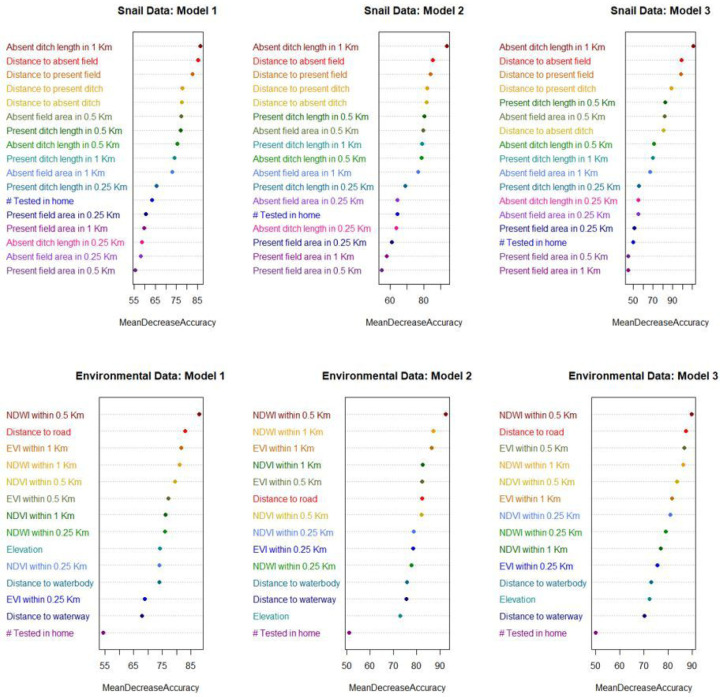
Variable importance plots for the snail and environmental data models.

The mean NDWI within 0.5 km of the home was the top predictor in all open-source environmental data models, resulting in a three-model summary score of 30 (Table 4 and Figure 4). Distance to the nearest road and the mean NDWI within 1 km of the home were the next most important predictors, each with a summary score of 23. EVI within 1 km and 0.5 km of the home were also ranked in the top five predictors, followed by NDVI at 0.5 km and NDVI at 1 km, which were ranked 6^th^ and 7^th^, respectively. None of the variables that used a 0.25 km buffer around the home was ranked in the top 50% of predictors, nor was elevation, the distance to waterbodies or waterways, or the number of people tested per household.

For each of the three models generated with the snail data and the environmental data, variable importance was determined using Mean Decrease in Accuracy (MDA). Each variable is assigned one color across all three models such that color can be used to highlight major shifts in variable importance ranks between models.

After dividing the snail habitat data (top), and the environmental data (bottom) 75:25 for training and validation, three balanced training datasets were obtained for each by oversampling the minority outcome class. These balancing repetitions were used to assess the stability of model performance metrics and variable importance rankings that results from using an oversampling approach to creating a balanced training dataset. After tuning each model using ten-fold cross-validation, the final models were run on the reserved testing data to generate model performance metrics and variable importance summaries (indicated by the Mean Decrease in Accuracy (MDA)). The ten predictors with the highest MDA in each model were given a score of 10 – 1 (10 being the score of the predictor with the highest MDA). Variable scores were then summed across the three models to create a three-model summary score of 30 – 0, 30 being the highest score possible (ranked first in all three models), while a score of 0 indicates that the variable was not ranked in the top ten in any of the three models. In this table, the top ~50% of predictors (determined by the three-model summary score) are shown above the dotted line in black, while those that were in the bottom 50% are below the dotted line and shown in gray.

The total length of all absent ditches (i.e., ditches where no snails were found) within 1 km of the home was the top predictor for all three snail models, followed by the distance to the nearest absent field, the distance to the nearest present field, and the distance to the nearest present ditch, respectively (Table 4, Figure 4). Like what was found with the environmental data models, none of the variables that used the smallest buffer size (0.25 km) around each home to summarize the snail habitat were ranked among the top 50% of predictors in the three-model summary score.

### Logistic Regressions and Predictions

In our simple logistic regression analyses for the environmental predictors, we found that the total distance to the nearest road was the only predictor that was ranked among the top 50% of predictors that was also significantly (p-value <0.05) associated with household *S. japonicum* infection status (Table 5). For each 1 km increase in the distance between the home and the nearest road, the log odds of household infection increased by 1.30 (standard error = 0.60, p-value = 0.03). NDWI and EVI within 0.5 km and 1 km of the home were positively associated with household infection status, whereas NDVI was negatively associated with infection status. The logistic regression results suggest a nonlinear relationship between household infection status and NDWI, NDVI, and EVI within 0.5 – 1 km of the home. An exploratory examination of lowess plots between the log odds of household infection status and NDWI, NDVI and EVI suggested threshold points, as the approximate lower quartile of NDWI (~< −0.2) and the upper quartile of NDVI (~>0.2) was associated with a lower log odds of infection than other values (data not shown). Although neither distance to the nearest waterway nor elevation were ranked among the top 50% of the environmental predictors, both were strongly negatively associated with household infection status (p-value <0.01).

For the snail predictors, the distance from the home to the nearest field where snails were present, the total length of present ditches within 0.5 km of the home, and the total area of absent fields within 0.5 km of the home were among the top 50% of predictors and were also linearly associated with household *S. japonicum* infection status (p-value <0.01). For each 1 km increase in the distance between a field where snails were present and the home, the log odds of household infection increased 0.92 (SE=0.33, p=0.005). Likewise, for each 0.1 km^2^ increase in fields where no snails were found within 0.5 km of the home, the log odds of household infection decreased 2.78 (SE=1.05, p=0.008). For every 1 km increase in the length of ditches where snails were found within a 1 km radius of the home, the log odds of household infection increased 0.78 (SE=0.26, p=0.002). Of those snail variables that were significantly (p<0.05) associated with household infection, fields were associated with a lower risk of infection, while ditches were associated with an increased risk of infection.

**Figure 5:**
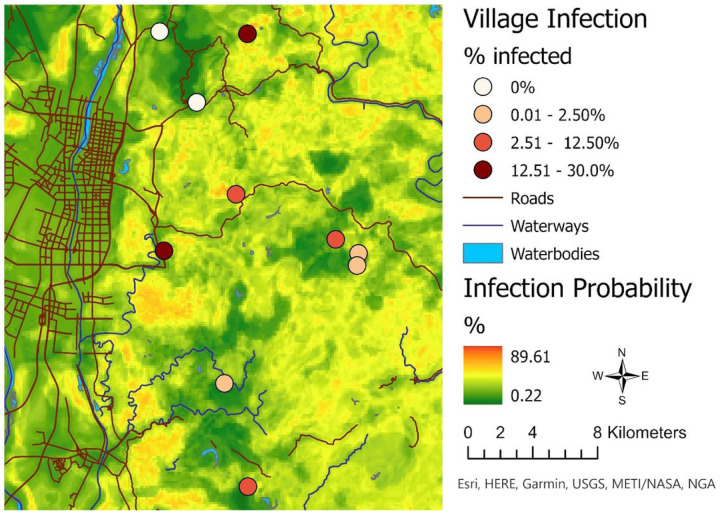
Prediction map showing the probability of *S. japonicum* infection using the top-performing environmental data model. The final top performing model was defined as the one with the highest kappa, accuracy, and receiver operating characteristic (ROC) area under the curve (AUC), respectively. Model performance metrics (Cohen’s kappa and accuracy) highlighted that the open-source environmental data models outperformed the snail data models. The top performing environmental data model was used to create a prediction surface of the probability of *S. japonicum* infection across the entire study area.

Given that the kappa and accuracy of the three final environmental models was higher than the kappa and accuracy of all the snail habitat models, the environmental model with the highest ROC AUC (Model 1; see Table 3) was used as our final prediction model. Using the final model, we generated a prediction surface for the entire study area to illustrate the predicted probability of infection across different landscapes within the study area (Figure 5). The predicted probability of infection for the study area ranged from 0.2% to 89.6%.

## Discussion

In this study, we set out to gain a better understanding of the strengths and limitations of on-the-ground-surveillance as compared to remote sensing and open-source environmental data for identifying pockets of schistosomiasis in a region approaching elimination. In our analysis, we found that the open-source environmental data models outperformed the snail data models in predicting household *S. japonicum* infection status in rural farming communities in Sichuan, China. Across our models, the sensitivity, specificity, NPV, PPV, kappa and overall accuracy of the environmental data models was higher than the snail data models. This has important implications. Whereas snail surveys are labor-intensive and time-consuming pursuits, the data from the environmental predictors models are readily available and free to download from the OpenStreetMap Contributors (30), JAXA EORC (32), and the USGS EROS Center’s image library (33). For the purposes of estimating local infection risk in areas approaching elimination, the ultimate payoff of investing resources into snail surveys may be lower than what could be achieved by limiting field activities to the production of comprehensive high-quality human and animal infection surveys, and investing in trained GIS professionals who can leverage open-source environmental data. Although hiring and training local cadres of GIS professionals with access to and proficiency in software designed for performing geospatial analyses would inevitably involve upfront costs, once obtained, this highly skilled and specialized workforce could repeat this analysis in other areas to fine-tune these models for use in other geographic regions and contexts and evaluate the generalizability of the findings presented in this study. What’s more, the analytical methods presented in this study could also be broadly applied by GIS professionals to a range of emerging or reemerging diseases across different landscapes and ecosystems in China and beyond in order to improve the broader understanding of the environmental conditions that can promote or interrupt the transmission of environmentally-mediated diseases.

As more locations across China approach elimination goals and *S. japonicum* becomes increasingly rare, intensive prevention and control programs and their *S. japonicum*-dedicated teams are likely to be phased out in favor of targeted surveillance and response methods. It is therefore becoming increasingly important to explore a range of lower-input alternatives to large-scale snail surveys for monitoring *S. japonicum* risk in the years to come. In this study, the low false positive rate of our environmental models (specificity = 0.951), suggests that open-source environmental data can serve as an effective alternative to large-scale snail surveys for ruling in the possibility of *S. japonicum* infection at fine spatial scales in areas on the verge of elimination. This is useful in the context of resource-limited control programs, in that it can serve as a first step in identifying areas where infections are likely to be present (and, conversely, ruling out areas where infections are unlikely to be found). This can enable the direction of resources such as infection screening, preventative prophylaxis and improved sanitation to areas that are predicted to have high infection probability and avoid diverting resources to regions where infections are unlikely to be present.

When looking at the relative variable importance of the open-source environmental data models, the three-model summary score highlighted NDWI within 0.5 km of the home, distance to the nearest road, and NDWI within 1 km of the home as the first, second and third best environmental predictors of household *S. japonicum* infection, respectively. Homes that were further from a road were significantly more likely to have one or more *S. japonicum* infection. This finding is consistent with the results of other studies, which have suggested that schistosomiasis infection risk is higher in areas that are further from a city (48, 49), a phenomenon potentially related to lower access to healthcare in more remote locations, as has been suggested elsewhere (50). In this study, NDWI was positively associated with infection risk, with some evidence of a threshold effect. This suggests that residents in homes situated in areas with more surface water nearby (within 1 km) have a greater risk of *S. japonicum* infection – an association that could be due to increased opportunities for human exposure to schistosomes through water contact, as has been previously found (9, 19, 49, 51–54). In a similar vein, we found that homes that were closer to waterways, as well as those at lower elevations were more likely to have *S. japonicum* infection than those that were nearer to waterways or situated at higher elevations. In the case of elevation, the negative association with household *S. japonicum* infection could potentially be linked to water accumulation at lower elevations, or a greater risk of encountering *O. hupensis* snails at lower elevations, as has been found in other studies (17, 28, 37). Taken together, the fact that the distance to the nearest waterway and household elevation were strong predictors of household infection, and that NDWI within 0.5 km and NDWI within 1 km of the home were the first and third best predictors in our RF models is consistent with what is known about the important role of water in the schistosomiasis transmission cycle and highlights the utility of using measures of surface water accumulation as a simple means of schistosomiasis risk characterization and surveillance.

In this study, NDVI was negatively associated with household infection such that households in the highest quartile of NDVI (~ > 0.2) had lower infection risk. Meanwhile, both the highest and lowest quartiles of EVI had lower infection risk than the middle two quartiles. Given that the EVI is particularly sensitive to canopy health (38), while NDVI tends to measure lower-lying crop health (41), our findings suggest that areas with moderate levels of canopy cover and lower levels of crop vegetation are at higher risk of *S. japonicum* infection. As far as we are aware, these results have not been replicated elsewhere, warranting further investigation into this phenomenon and its potential underlying mechanisms.

While the models using snail survey data did not perform as well as the open-source environmental data models, we identified a few key predictors that shed light on the relationship between snail habitat and human infections. First, proximity to and the total length of ditches in the area surrounding the home (0.5–1 km radius) were consistently among the top predictors of household *S. japonicum* infection and generally followed the anticipated direction of association. For example, in the case of ditches where snails were present, our simple logistic regression models suggest that homes that were closer to or those with a greater density of ditches where snails were present were more likely to have one or more residents with *S. japonicum* infection. This aligns with our expectations, as more snail habitat sites near the home would be expected to correspond with an increasing number of opportunities to encounter infected snails and become infected. Second, we found surprising evidence that fields may be protective against *S. japonicum* infection – greater density of fields near the home where snails were present or absent, and proximity to fields where snails were present were all associated with decreased household *S. japonicum* infection risk. While determining why this might be the case was beyond the scope of this study, we hypothesize that it is related to a lower overall density of snails across fields, as compared to ditches where snails are likely more compactly situated.

We assessed which spatial scales were most relevant to household *S. japonicum* infection risk by applying three different buffer sizes (0.25 km, 0.5 km, 1 km) around the home to summarize each of our four main snail habitat predictor categories (present fields, absent fields, present ditches, present ditches), and our three environmental indexes measuring surface water and vegetation (NDWI, NDVI, EVI). For all models, only those predictors that used a 0.5 or 1 km buffer were among the top 50% of predictors according to our three-model summary score. Although other studies from China have also highlighted the importance of aggregated or village-scaled measures of *S.japonicum* risk (55, 56), this is an important consideration in light of a push for precision mapping of schistosomiasis. We found that some of the strongest predictors of a high-resolution outcome (household-level infection) were characteristics of the neighborhood rather than the area immediately surrounding the home.. Overall, this highlights the important role that spatial scales can play when assessing predictors of environmentally-mediated diseases like schistosomiasis. As a result, we suggest that future studies and interventions focused on the proximity of the home to snail habitats or high-risk environmental features will benefit from considering a range of potential scales of influence, rather than focusing solely on the immediate surroundings of the home.

There are a few limitations to this analysis that warrant further discussion. First, we had a relatively small sample size (N = 283 households), and many predictors in each of our models (N = 17 in the snail data models, and N = 14 in the open-source environmental data models). While RF models are wellrecognized for being robust to small sample sizes and large predictor sets (57), smaller samples result in reduced power to detect rare events and an increased risk that the sample is unrepresentative of the underlying population. We compensated for this, in part, by running multiple models and summarizing broad-scale trends in performance and variable rankings that held across multiple iterations of model building. Additionally, because our focus was on fine-scale prediction, the relatively small study area of interest (~ 700 km^2^) resulted in the exclusion of certain predictors (e.g., temperature, and precipitation). With a maximum distance of < 25 km between any two households, weather would not be expected to vary substantially, and therefore was not considered in this study. Furthermore, the relatively small geographic area of this study tends to restrict the generalizability of our findings to those areas with similar ecosystems and climates (hilly regions of China), and those that use similar snail survey techniques to those used in this study.

Another limitation in this analysis was the class imbalance for the outcome variable, as misclassification rates tend to increase when using RF models to predict outcomes that do not have roughly equal numbers of observations within each category (58). Overall, 39/283 (13.8%) households had one or more cases of schistosomiasis. To account for the high degree of class imbalance in our outcome, we oversampled the minority class in the training datasets. However, for the reserved validation dataset, the class imbalance remained, resulting in inflated accuracy measures. As such, we recommend that readers prioritize the kappa statistic over the accuracy measure when considering the performance of our models, as this was developed to help correct for bias due to class imbalance (44). Another noteworthy limitation in this analysis is that the variable importance measures were likely impacted by the high degree of correlation between some of our predictors (e.g., two different measures of vegetation, or the three different spatial scales used to develop predictors), as the variable importance rankings that are used in RF models become less reliable when predictors are highly correlated with one another (59). As such, the relative rankings of predictors should be interpreted with caution, instead looking at broad-scale trends in predictor rankings (e.g., ranked in the top 50% of predictors, versus the bottom 50% of predictors). Finally, it is worth noting that snail survey data is inherently incomplete, as it is inevitable that not every snail is going to be detected in each field, ditch or other environmental feature and snail surveys provide only a snapshop of highly dynamic snail populations. The snail surveys were conducted using standard protocols (27), meaning that the data on snail habitats is likely to represent typical data for the area, making our analysis an assessment of the predictive capacity of real-world snail data.

## Conclusion

In this study, we compared the use of labor-intensive snail survey data with that of open-source environmental data for developing prediction models aimed at predicting household infection status among rural farming communities in China. Overall, we found that freely available environmental data can be used to predict household infection status among rural farming communities in Sichuan Province, China, with high accuracy. Furthermore, the open-source environmental data ultimately outperformed the snail habitat data, suggesting that, prior to conducting comprehensive snail surveys, the overarching goal of the surveys ought to be considered to determine whether less resource-intensive methods might be suitable. Not only has this analysis helped to improve our understanding of where and when transmission is most likely to be occurring in the study area, but it has also highlighted specific aspects of the local environment that are associated with household infection – for example, homes that are furthest from roads, or those surrounded by more surface water – which can become the target of future surveillance and control efforts. Ultimately, by expanding the current body of knowledge on the utility of using open-source environmental data for predicting infection risks as well as some of the limitations and uses of snail survey data in the context of household risk characterization, this study provides valuable insight on priority locations and corresponding tailored control activities that can be used to maximize the impact of surveillance and intervention efforts in in areas approach elimination.

## Figures and Tables

**Table 1:** List of predictors generated for each model.

**Snail survey data model**	**Environmental predictors model**
Presence sites (one or more snails found)	Open data
**Ditches where snails were present**	**Built and natural environment**
Distance from home to the nearest identified present ditch (km)	Distance to nearest waterway (km)
Total length of present ditches within 0.25 km radius of the home (km)	Distance to nearest waterbody (km)
Total length of present ditches within 0.5 km radius of the home (km)	Distance to nearest road (km)
Total length of present ditches within 1 km radius of the home (km)	Elevation (m)
**Fields where snails were present**	
Distance from home to the nearest identified present field (km)	Remotely sensed data
Total area of present fields within 0.25 km radius of the home (km^2^)	**Normalized Difference Water Index (NDWI)**
Total area of present fields within 0.5 km radius of the home (km^2^)	Average NDWI within 0.25 km radius of the home
Total area of present fields within 1 km radius of the home (km^2^)	Average NDWI within 0.5 km radius of the home
	Average NDWI within 1 km radius of the home
Absence sites (no snails found)	**Normalized Difference Vegetation Index (NDVI)**
**Ditches where snails were absent**	Average NDVI within 0.25 km radius of the home
Distance from home to the nearest identified absent ditch (km)	Average NDVI within 0.5 km radius of the home
Total length of absent ditches within 0.25 km radius of the home (km)	Average NDVI within 1 km radius of the home
Total length of absent ditches within 0.5 km radius of the home (km)	Enhanced Vegetation Index (EVI)
Total length of absent ditches within 1 km radius of the home (km)	Average EVI within 0.25 km radius of the home
**Fields where snails were absent**	Average EVI within 0.5 km radius of the home
Distance from home to the nearest identified absent field (km)	Average EVI within 1 km radius of the home
Total area of absent fields within 0.25 km radius of the home (km^2^)	
Total area of absent fields within 0.5 km radius of the home (km^2^)	Other predictors included in the models
Total area of absent fields within 1 km radius of the home (km^2^)	Number of people tested in the household (N)
Other predictors included in the models	
Number of people tested in the household (N)	
NDWI = Normalized Difference Water Index	
NDVI = Normalized Difference Vegetation Index	
EVI = Enhanced Vegetation Index	

**Table 2.** Summary of household variables.

Shared model characteristics	N	%		
**Number of infections per household**				
0	244	86.22		
1	33	11.66		
2+	6	2.12		
**Number of people tested per household**	.	.	.	.
1	83	29.33		
2	146	51.59		
3	42	14.84		
4+	12	4.24		
Snail models	Mean	SD	Min.	Max.
**Ditches**				
Distance from home to nearest present ditch (km)	0.57	0.89	<0.01	3.26
Length of present ditches within 0.25 km of the home (km)	0.24	0.28	0.00	0.97
Length of present ditches within 0.5 km of the home (km)	0.65	0.61	0.00	2.69
Length of present ditches within 1 km of the home (km)	1.74	1.70	0.00	7.31
Distance from home to nearest absent ditch (km)	0.26	0.23	<0.01	1.05
Length of absent ditches within 0.25 km of the home (km)	0.23	0.28	0.00	0.93
Length of absent ditches within 0.5 km of the home (km)	0.77	0.65	0.00	2.11
Length of absent ditches within 1 km of the home (km)	2.22	1.35	0.00	6.80
**Fields**				
Distance from home to nearest present field (km)	0.58	0.51	<0.01	1.55
Area of present fields within 0.25 km of the home (km^2^)	0.01	0.01	0.00	0.05
Area of present fields within 0.5 km of the home (km^2^)	0.02	0.04	0.00	0.13
Area of present fields within 1 km of the home (km^2^)	0.04	0.06	0.00	0.16
Distance from home to nearest absent field (km)	0.36	0.54	<0.01	2.20
Area of absent fields within 0.25 km of the home (km^2^)	0.01	0.01	0.00	0.19
Area of absent fields within 0.5 km of the home (km^2^)	0.02	0.02	0.00	0.09
Area of absent fields within 1 km of the home (km^2^)	0.06	0.06	0.00	0.04
Environmental data models	Mean	SD	Min.	Max.
**Built and natural environment**				
Distance from home to the nearest waterway (km)	3.02	1.82	0.06	5.44
Distance from home to the nearest waterbody (km)	2.11	0.95	0.37	3.91
Distance from home to the nearest road (km)	0.36	0.26	<0.01	1.27
Elevation of the home (m)	573.45	54.81	495.0	685.0
**Remotely sensed data**				
Mean NDWI within 0.25 km of the home	−0.19	0.02	−0.23	−0.15
Mean NDWI within 0.5 km of the home	−0.19	0.02	−0.21	−0.16
Mean NDWI within 1 km of the home	−0.19	0.01	−0.20	−0.17
Mean NDVI within 0.25 km of the home	0.18	0.02	0.14	0.23
Mean NDVI within 0.5 km of the home	0.18	0.02	0.14	0.22
Mean NDVI within 1 km of the home	0.18	0.02	0.15	0.21
Mean EVI within 0.25 km of the home	0.40	0.03	0.36	0.48
Mean EVI within 0.5 km of the home	0.40	0.02	0.37	0.47
Mean EVI within 1 km of the home	0.40	0.02	0.38	0.46

NDWI = Normalized difference Water Index

NDVI = Normalized difference vegetation Index

EVI = Enhanced vegetation Index

**Table 3.** Performance metrics for the snail and environmental data models.

Performance metrics						
	Snail survey data models			Open-source environmental data models		
	Model 1	Model 2	Model 3	Model 1	Model 2	Model 3
AUC	0.852	0.849	0.843	0.800	0.784	0.798
Accuracy	0.845	0.859	0.845	0.887	0.887	0.887
Accuracy 95% CI	0.74 – 0.92	0.76 – 0.93	0.74 – 0.92	0.79 – 0.95	0.79 -- 0.95	0.79 -- 0.95
NIR^a^	0.859	0.859	0.859	0.859	0.859	0.859
P-Value (Accuracy > NIR)	0.706	0.583	0.706	0.316	0.316	0.316
Kappa^b^	0.332	0.365	0.332	0.492	0.492	0.492
Sensitivity	0.4	0.4	0.4	0.5	0.5	0.5
Specificity	0.918	0.934	0.918	0.951	0.951	0.951
Pos Pred Value	0.444	0.5	0.444	0.625	0.625	0.625
Neg Pred Value	0.903	0.905	0.903	0.921	0.921	0.921

a No Information Rate

b Due to the high degree of imbalance between the outcome classes across the study period, the Cohen’s kappa statistic is a useful metric for our models, as it helps to correct bias that results when rewarding the prediction of the majority class. The benchmark values outlined by Landis & Koch (1977) are useful here for determining the relative strength of the predictive models: <0.00 = Poor; 0.00 – 0.20= Slight; 0.21 – 0.40 = Fair; 0.41 – 0.60 = Moderate; 0.61 – 0.81 = Substantial; 0.81 – 1.0 = Almost Perfect.

**Table 4.** Summary of variable importance rankings for the snail and environmental data models.

	Model 1:	Model 2:	Model 3:	Three-model summary:
	Variable score	Variable score	Variable score	Final variable score
**Snail model predictors**				
Absent ditch length within 1 Km	10	10	10	30
Distance to absent field	9	9	9	27
Distance to present field	8	8	8	24
Distance to present ditch	7	7	7	21
Distance to absent ditch	6	6	4	16
Present ditch length within 0.5 Km	4	5	6	15
Absent field area within 0.5 Km	5	4	5	14
Absent ditch length within 0.5 Km	3	2	3	8
Present ditch length within 1 Km	2	3	2	7
Absent field area within 1 Km	1	1	1	3
Present ditch length within 0.25 Km	0	0	0	0
# People tested in the home	0	0	0	0
Present field area within 0.25 Km	0	0	0	0
Present field area within 1 Km	0	0	0	0
Absent ditch length within 0.25 Km	0	0	0	0
Absent field area within 0.25 Km	0	0	0	0
Present field area within 0.5 Km	0	0	0	0
				
**Environmental model predictors**				
NDWI within 0.5 Km	10	10	10	30
Distance to the nearest road	9	5	9	23
NDWI within 1 Km	7	9	7	23
EVI within 1 Km	8	8	5	21
EVI within 0.5 Km	5	6	8	19
NDVI within 0.5 Km	6	4	6	16
NDVI within 1 Km	4	7	2	13
NDVI within 0.25 Km	1	3	4	8
NDWI within 0.25 Km	3	1	3	7
EVI within 0.25 Km	0	2	1	3
Elevation	2	0	0	2
Distance to nearest waterbody	0	0	0	0
Distance to nearest waterway	0	0	0	0
# People tested in the home	0	0	0	0

**Table 5.** Simple logistic regression results.

Snail Habitat Data Models			
			
Scaled predictors^a^	PE ^b^	SE ^c^	P-value
Absent ditch length within 1 Km	0.17	0.13	0.179
Distance to absent field	−0.28	0.37	0.450
Distance to present field	0.92	0.33	0.005**
Distance to present ditch	−0.42	0.27	0.125
Distance to absent ditch	0.05	0.75	0.944
Present ditch length within 0.5 Km	0.78	0.26	0.002**
Absent field area within 0.5 Km	−2.78	1.05	0.008**
Absent ditch length within 0.5 Km	−0.08	0.27	0.757
Present ditch length within 1 Km	0.32	0.08	<0.001**
Absent field area within 1 Km	−0.75	0.34	0.029*
Present ditch length within 0.25 Km	0.81	0.58	0.164
# People tested in the home	0.37	0.19	0.049*
Present field area within 0.25 Km	−7.83	3.59	0.029*
Present field area within 1 Km	−0.97	0.43	0.023*
Absent ditch length within 0.25 Km	0.01	0.60	0.981
Absent field area within 0.25 Km	−5.02	2.88	0.081
Present field area within 0.5 Km	−1.52	0.73	0.036*

Open-Source Environmental Data Models			

Scaled predictors^a^	PE ^b^	SE ^c^	P-value
NDWI within 0.5 Km	1.33	1.09	0.221
Distance to the nearest road	1.30	0.60	0.029*
NDWI within 1 Km	1.03	1.31	0.432
EVI within 1 Km	0.88	0.78	0.261
EVI within 0.5 Km	1.22	0.65	0.059
NDVI within 0.5 Km	−0.86	0.85	0.311
NDVI within 1 Km	−0.48	0.99	0.632
NDVI within 0.25 Km	−0.84	0.73	0.250
NDWI within 0.25 Km	1.23	0.93	0.184
EVI within 0.25 Km	1.11	0.56	0.050*
Elevation	−0.13	0.04	<0.001**
Distance to nearest waterbody	−0.07	0.18	0.706
Distance to nearest waterway	−0.29	0.10	0.003**
# People tested in the home	0.37	0.19	0.049*

a For both the snail habitat data (top), and the environmental predictors data (bottom), simple logistic regression models were run to determine the direction of association with household *S. japonicum* infection status. Each predictor was scaled to make a one-unit change represent meaningful incremental changes. The units used for each snail variable are as follows: for the distance to the nearest present ditch, absent ditch, present field and absent field, the unit of change was 1 km; for the total present ditch length and total absent ditch length within 0.25 km, 0.5 km and 1 km of the home, the unit of change was 1 km; for the area of present fields and area of absent fields within 0.25 km, 0.5 km and 1 km of the home, the unit of change was 0.1 km^2^; the unit of change was 1 person. The units used for each environmental variable are as follows: for NDWI, NDVI and EVI, the unit of change was 0.1 (index range of −1 to +1); for the distance to the nearest road, waterway or waterbody, the unit of change was 1 km; for elevation, the unit of change was 10 meters; for the number of people tested in the home, the unit of change was 1 person.

## Data Availability

The datasets used in this analysis have not been made publicly available because they correspond with household coordinates and private health information of study participants; there data will not be made publicly available for confidentiality reasons.

## Abbreviations

NDWI: Normalized Difference Water Index
NDVI: Normalized Difference vegetation Index
GPS: Global Positioning System
GIS: Geographic Information System
JAXA: Japan Aerospace Exploration Agency
EORC: Earth Observation Research Center
ALOS: Advanced Land Observing Satellite
USGS: United States Geological Survey
EROS: Earth Resources Observation and Science Center
NASA: National Aeronautics and Space Administration
MODIS: Moderate Resolution Imaging Spectroradiometer
EVI: Enhanced vegetation Index
NIFR: Near Infrared
RF: Random Forests
ROC: Receiver Operator Curve
AUC: Area Under the Curve
PPV: Positive Predictive Value
NPV: Negative Predictive Value
NIR: No Information Rate
MDA: Mean Decrease in Accuracy
SD: Standard Deviation
